# Uterine Fundus Remodeling after Hysteroscopic Metroplasty: A Prospective Pilot Study

**DOI:** 10.3390/jcm10020260

**Published:** 2021-01-12

**Authors:** Paolo Casadio, Giulia Magnarelli, Mariangela La Rosa, Andrea Alletto, Alessandro Arena, Enrico Fontana, Ciro Morra, Maria Rita Talamo, Matilde Fabbri, Kevin Giovannico, Agnese Virgilio, Diego Raimondo, Francesca Guasina, Roberto Paradisi, Renato Seracchioli

**Affiliations:** 1 Gynecology and Human Reproduction Physiopathology Unit, IRCCS Azienda Ospedaliero-Universitaria di Bologna, 40138 Bologna, Italy; paolo.casadio@aosp.bo.it (P.C.); mariangela.larosa@studio.unibo.it (M.L.R.); andrea.alletto@studio.unibo.it (A.A.); alessandro.arena6@unibo.it (A.A.); enrico.fontana2@studio.unibo.it (E.F.); matilde.fabbri5@studio.unibo.it (M.F.); kevin.giovannico@studio.unibo.it (K.G.); agnese.virgilio@studio.unibo.it (A.V.); diego.raimondo2@unibo.it (D.R.); roberto.paradisi@unibo.it (R.P.); renato.seracchioli@aosp.bo.it (R.S.); 2 Department of Gynecology and Obstetrics, Maggiore Hospital, 40133 Bologna, Italy; ciro.morra@ausl.bo.it (C.M.); mariarita.talamo@ausl.bo.it (M.R.T.); 3 Department of Gynecology and Obstetrics, Santa Chiara Regional Hospital, 38122 Trento, Italy; francesca.guasina@apss.tn.it

**Keywords:** septate uterus, uterine malformation, uterine fundus, hysteroscopy, ultrasound, remodeling

## Abstract

The septate uterus is the most common congenital uterine malformation and is treated by hysteroscopic metroplasty. There are few studies on the fundal uterine changes that occur after surgery. We designed a pilot prospective observational study to evaluate by three-dimensional transvaginal ultrasound (3D-TVS) the changes not only of the internal fundal uterine profile, but also of the external one, after hysteroscopic metroplasty. Sixty women who underwent hysteroscopic metroplasty for partial or complete uterine septum (U2a and U2b subclasses of ESHRE/ESGE classification) were enrolled. We performed 3D-TVS after surgery confirming optimal removal of the septum. However, at ultrasound follow-up after three months, we observed a significant increase (*p* < 0.001) in the residual septum (Zr) (3.7 mm (95% CI: 3.1–4.4)), the myometrial wall thickness (Y) (2.5 mm (95% CI: 2.0–3.0)) and the total fundal wall thickness (Y + Zr) (6.2 mm (95% CI: 5.5–6.9)). Forty-three patients (72%) required a second step of hysteroscopic metroplasty. Moreover, the shape of uterine fundus changed in 58% of cases. We actually observed a remodeling of the uterine fundus with modifications of its external and internal profiles. Therefore, we propose to always perform a second ultrasound look at least three months after the metroplasty to identify cases that require a second- step metroplasty.

## 1. Introduction

Congenital uterine malformations result from abnormal development, fusion or resorption of the Mullerian ducts during prenatal growth. The septate uterus is the most common uterine abnormality, accounting for 35% of all identified uterine malformations [1]. It has an estimated prevalence of 2–3% in women of reproductive age [2,3,4].

According to the recent classification proposed by the CONgenital UTerine Anomalies (CONUTA) European Society of Human Reproduction and Embryology (ESHRE)/European Society for Gynaecological Endoscopy (ESGE) Working Group [5], a septate uterus is defined as a congenital uterine anomaly with pathological persistence of the median septum due to an abnormal resorption phenomenon, a normal outline of the uterine fundus and a median internal fundal indentation exceeding 50% of the uterine wall thickness regardless of the length of the septum (class U2 of ESHRE/ESGE classification) [5,6]. This category is further divided into two subclasses (U2a and U2b), depending on whether the apex of the septum reaches the internal cervical os or stops above it. The internal indentation has to be measured as the distance between the interostial line and the edge of the inner indentation, while the uterine wall thickness is the distance between the interostial line and the uterine serosa [6].

Septate uterus is more frequent in infertile patients and some evidence suggests that it may increase the risk of miscarriage, preterm delivery and fetal malpresentation [7]. The pathophysiology underlying the poor obstetric outcomes is not yet known, but the different histological composition of the endometrium lining the septum could explain the impaired reproductive outcome of embryos implanted in the intrauterine septum [2].

Therefore, the rationale behind the septum incision is based on restoring a regular morphology of the uterine cavity by removing the tissue that is not suitable for embryo implantation. In the past, it was performed by laparotomic hysterotomy [8], but in the 1970s Edström and Fernström [9,10] attempted to remove the uterine septum by hysteroscopy and this approach is currently considered the first-line surgical therapy and is a common practice in many countries [11].

Metroplasty is still a matter of debate, because the data available in literature are based only on observational studies that frequently suggest an improvement in reproductive performance after this surgical treatment. However, Rikken et al. recently published a retrospective multicenter cohort study comparing the expectant management with surgery in women with septate uterus and desire for pregnancy [12]. They concluded that hysteroscopic metroplasty does not increase the chance of live birth nor decrease the chance of pregnancy loss or preterm birth. According to some authors, these results are not supported by valid evidence, due to numerous methodological limitations and biases (i.e., multiple definitions of uterine septum and non-homogeneous metroplasty technique) [13].

In conclusion, there is little evidence to draft guidelines and propose a standardized hysteroscopic metroplasty. A randomized controlled trial (RCT) is still needed on this topic [14,15,16,17,18,19,20,21,22]. The 3D-transvaginal sonography (3D-TVS) is currently the most widely used method for the initial study of the uterine morphology and can also be performed to study/evaluate the outcomes of hysteroscopic metroplasty [23,24]. To date, few studies has shown the modifications of the profile of uterine cavity after hysteroscopic metroplasty [25,26]. Instead, to our knowledge, this is the first work that analyzes the uterine fundal morphology before and after hysteroscopic treatment.

The first aim of this study is to evaluate the modifications of the inner and outer uterine profiles after hysteroscopic metroplasty, using 3D-TVS performed three months after surgery. The second endpoint is to evaluate the potential need for a second metroplasty surgical procedure.

## 2. Materials and Methods

### 2.1. Subjects

Our prospective, observational study was conducted at the Hysteroscopy Unit of the Department of Gynecology and Physiopathology of Human Reproduction, S. Orsola—Malpighi Hospital, University of Bologna, Italy. The study was approved by the local ethics committee (205/Oss/AOUBo).

Between October 2017 and June 2020, we enrolled 60 women desiring pregnancy, with partial or complete uterine septum (classes U2a and U2b according to the ESHRE/ESGE classification system) [6] diagnosed by 3D-TVS and confirmed by outpatient hysteroscopy. Between October 2017 and June 2020, 60 women with a history of ≥1 miscarriage or infertility and desiring pregnancy, with a 3D-TVS diagnosis of partial or complete uterine septum (classes U2a and U2b according to the ESHRE/ESGE classification system) [6] and an outpatient hysteroscopic confirmation, were enrolled in the study. All patients with diagnosis of infertility sought pregnancy for over 12 months and their partner’s sperm was normal for count, mobility and morphology of spermatozoa. As inclusion criteria, all the women included in the study reported a history of ≥1 miscarriage or infertility. Exclusion criteria were ongoing pregnancy (confirmed by a positive preoperative beta-HCG), malignancy, previous uterine surgery, presence of other intrauterine pathologies (i.e., myomas, polyps, and intrauterine adhesions), complex Mullerian malformations and other severe comorbidities (coagulation disorders, systemic disease, and serious cardiac disease). We advised all enrolled women to stop any contraception to avoid possible bias related to the evaluation of presurgical measurements and postsurgical outcomes.

All women underwent 3D-TVS performed by a single experienced operator (G.M.) during the mid-luteal phase of the cycle (days 21–25), which is considered the optimal time to study the uterine cavity [27]. During this phase, the endometrium is thick and echogenic, making it possible to differentiate the uterine cavity from the surrounding myometrium in the reconstruction of the uterus on the coronal plane. The 3D-TVS was performed using a Voluson E6 system (GE Healthcare, Little Chalfont, England) with a RIC 5–13 MHz volume transducer for all acquisitions. All volumes were then evaluated with dedicated software (4D View 14.4; GE Medical Systems) for offline analysis by an experienced investigator blinded to the clinical data (A.A.).

All measurements and evaluation of the uterine fundus profile were performed in the midcoronal plane of the uterus according to the methodology of Grimbizis et al. [6]. The uterine wall thickness (Y) was measured as the distance between the line connecting the tubal ostia (X) and the fundal uterine serosa, while any internal indentation (Z) was measured as the distance between the interostial line (X) and the edge of the indentation.

According to the ESHRE/ESGE classification system, the uterine septum was considered to be any internal indentation with a length that exceeds 50% of the uterine wall thickness and a normal outline. If the apex of the septum reached the internal uterine os, it was defined as complete; otherwise, it was considered partial [5,6].

### 2.2. Surgery

After the presurgical evaluation, hysteroscopic metroplasty was performed in an outpatient setting during the early follicular phase of the cycle. No pharmacologic preparations were administered to minimize the endometrium thickness. Hysteroscopic surgery was performed under paracervical anesthesia achieved by administrating 16 mL of Ropivacaine 7.5 mg/mL through four injections at the level of the vaginal fornixes, respectively, at three, five, seven and nine hours at the portio, corresponding to the lateral parametria and uterosacral ligaments. We used a 5 mm diameter continuous-flow hysteroscope with an oval profile, a 30° fore-oblique telescope, and a 5Fr operating channel (Office Continuous Flow Operative Hysteroscopy “size 5”; Karl Storz, Tuttlingen, Germany).

**Figure 1:**
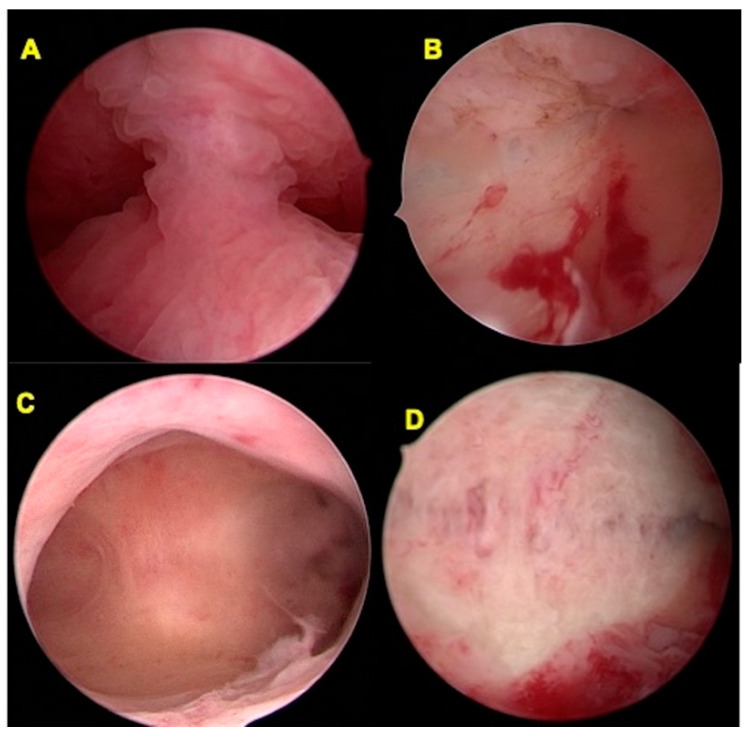
Hysteroscopic view of a complete and partial uterine septum before and after hysteroscopic metroplasty. (**A**) Hysteroscopic view of a complete uterine septum at the beginning of surgery; (**B**) Hysteroscopic view of the previous uterine cavity at the end of the metroplasty; (**C**) Hysteroscopic view of a partial complete uterine septum at the beginning of surgery; (**D**) Hysteroscopic view of the previous uterine cavity at the end of the metroplasty.

The uterine cavity was distended with saline solution (0.9% NaCl), supplied through an electronic system of irrigation and aspiration (Endomat, Karl Storz, Tuttlingen, Germany). We set a continuous flow control of 200–350 mL/min, the negative pressure suction at 0.2 bar and the positive pressure at 80–100 mmHg, obtaining an intrauterine pressure of about 40 mmHg throughout all the metroplasty. All surgical procedures were performed by the same expert surgeon (P.C.), according to the technique of Di Spiezio et al. [23], characterized by using a novel graduated intrauterine palpator (Karl Storz, Tuttlingen, Germany) (Figure 1).

The septum incision began at the apex with a straight bipolar electrode, setting the mildest vapor cutting mode (VC3) with a power of 50 W on the electrosurgical generator (Versapoint; Olympus, Hamburg, Germany). The electrode was used from one side of the septum to the other with latero-lateral movement and from apex to base along a median plane of the septum. After the removal of about three quarters of the septum, we replaced the bipolar electrode with miniature cold scissors to complete the septum incision until reaching the uterine fundus. Metroplasty was stopped when the graduated intrauterine palpator, introduced through the working channel of the hysteroscope, showed that the incised septum corresponded to the presurgical ultrasound measurements to obtain a total fundal uterine wall thickness of 1 cm ((Y + Z)—incised septum = 1 cm), as reported by Di Spiezio et al. [23]. At the end of the procedure, we applied a Hyaluronic acid derivative gel (Hyalobarrier Gel ENDO, Novus Pharma Solution) into the uterine cavity through the operative channel of the hysteroscope to reduce intrauterine adhesions.

**Figure 2:**
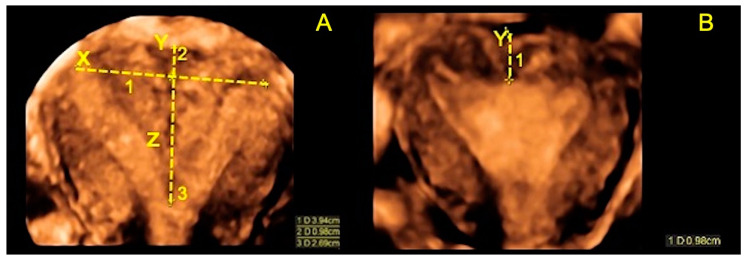
Ultrasound images of a complete septate uterus (U2b according to ESHRE/ ESGE) before and after surgery. (**A**) Ultrasound midcoronal view of the complete septate uterus before hysteroscopic metroplasty with all the measurements of the septum (the uterine wall thickness (Y), the base of the septum (X) and the length of the septum (Z)); (**B**) Ultrasound midcoronal view of the same uterus three hours after surgery. The fundal uterine wall thickness is 1 cm, confirming the complete incision of the septum.

On the same day as the surgical procedure, a 3D-TVS was performed to evaluate the uterine cavity and the thickness of the fundal uterine wall. In all cases, the uterine cavity appeared regular with a total fundal myometrial thickness of 1 cm, calculated as the sum of Y and the Z residual (Zr), measured as the distance between the interostial line and the new inner uterine profile. This confirmed the complete incision of the septum (Figure 2).

**Figure 3:**
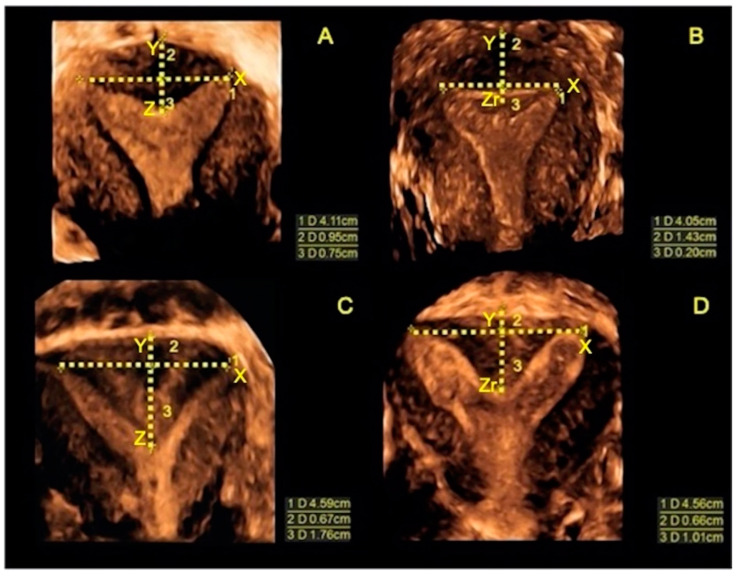
Ultrasound comparison between external and inner uterine profiles of septate uterus before surgery and three months after surgery. In each image, all the measurements of the septum are shown (the uterine wall thickness (Y), the base of the septum (X), the length of the septum before surgery (Z) and the length of the new inner uterine edge after surgery (Zr)). (**A**) Ultrasound midcoronal view of a partial septate uterus (U2a according to ESHRE/ ESGE) before surgery; (**B**) Ultrasound midocoronal image of the same uterus three months after surgery, characterized by the remodeling of the fundus; (**C**) Ultrasound midcoronal view of another partial septate uterus (U2a according to ESHRE/ ESGE) before surgery; (**D**) Ultrasound midcoronal image of the previous uterus three months after surgery, characterized by a residual partial septum. This patient was scheduled for a second- step metroplasty.

A transvaginal ultrasound was performed during the luteal phase of the third menstrual cycle after surgery. The uterine cavity was evaluated to identify cases requiring a second-step surgery. Moreover, the morphology of the uterine fundus (both internal and external profiles) was compared with that before surgery. We measured the distance between the interostial line and the new inner uterine edge (Zr), and the distance between the interostial line and the outer fundal contour (Y). According to the ESHRE/ESGE classification system, the inner fundal indentation was considered a residual septum only when the value of Zr exceeded 50% of the measure of Y [5] (Figure 3). In these cases, we considered the metroplasty incomplete only when Y + Zr was greater than 1 cm, scheduling for a second surgical step. In case of second- step metroplasty, outpatient hysteroscopy was performed in the early proliferative phase of the following menstrual cycle, cutting the residual uterine septum with cold miniature scissors, according to the technique described above.

### 2.3. Statistical Analysis

We used the estimates of Di Spiezio Sardo et al. [23] to obtain some of our study parameters. We hypothesized that the mean (±SD) length of the Zr at the end of optimal incision was 2.5 ± 1.25 mm, and the mean (±SD) length of Zr at three months follow-up was 3.5 ± 2.5 mm. Assuming a correlation between paired measurements of 0.5, the minimum number of optimal incisions required to detect the resulting delta of 1 mm with a power of 90% and a 5% significance level was 52 incisions. Hypothesizing an attrition rate of 10%, the overall sample size was increased to 57 incisions. The paired *t*-test was used to compare Z, Y and (Y + Z) values after surgery and at the three-month evaluation. Differences in the shape of the uterine fundus before surgery and three months after surgery were assessed with the Stuart–Maxwell test for marginal homogeneity.

We used the non-parametric Spearman’s rank correlation coefficient (r) to investigate the association of Z and (Y + Z) measured before hysteroscopic treatment with the increase in (Y + Zr) three months after surgery. Finally, the two-sample *t*-test was used to evaluate whether any changes in the shape of the uterine fundus after metroplasty were related to mean Z and (Y + Z) values measured before surgery.

The significance level was set at 5%. All analyses were carried out with Stata software, version 15 (StataCorp. 2017. Stata Statistical Software: Release 15. College Station, TX: StataCorp LLC, USA).

## 3. Results

In thirty-eight months, we enrolled sixty-three women of reproductive age, but three were excluded for not participating in the follow-up. Thus, sixty women entered the study. The main characteristics of the included women are summarized in Table 1.

In all women, hysteroscopic metroplasty was performed without any intraoperative or early post-operative complications. After the surgical procedure, the 3D-TVS showed a regular uterine cavity in all cases and the complete removal of the septum was always confirmed by a final fundal myometrial thickness of 1 cm.

**Figure 4:**
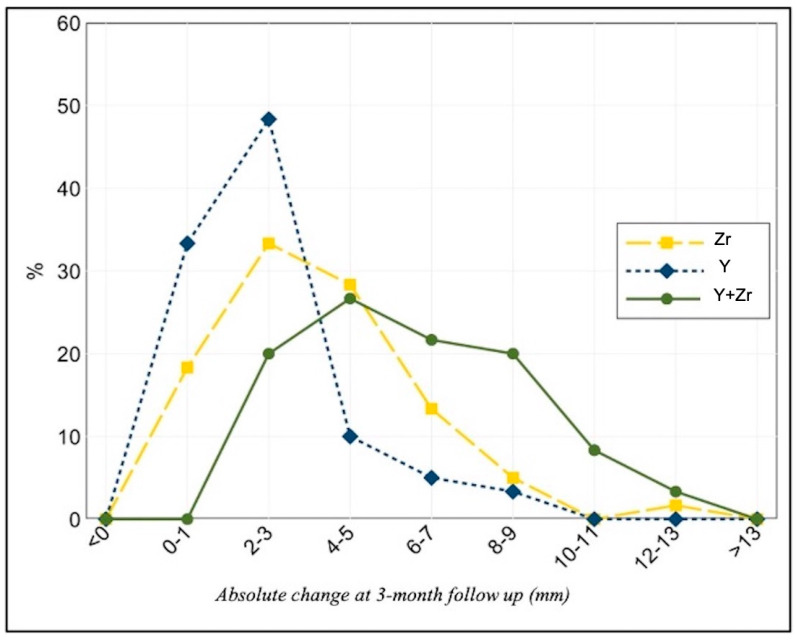
Distribution of absolute changes in Zr, Y and (Y + Zr) at three-month follow-up (*n* = 60).

At the three months follow-up, the total fundal uterine wall (Y + Zr) was thicker than that measured immediately after metroplasty due to the increase in both Y and Zr values. The mean increase in Zr, Y and (Y + Zr) at the three months follow-up was significantly different from zero (*p* < 0.001 for all measures). In particular, the mean increase in Zr, Y and (Y + Zr) was of 3.7 mm (95% CI: 3.1–4.4), 2.5 mm (95% CI: 2.0–3.0) and 6.2 mm (95% CI: 5.5–6.9), respectively. The percentage distribution of the absolute changes in the three measures is shown in Figure 4.

**Figure 5:**
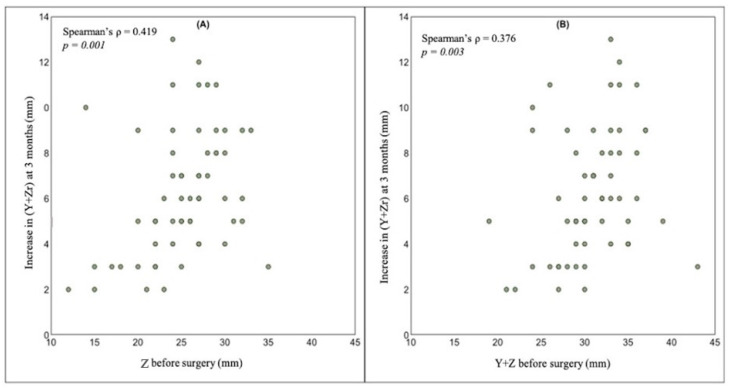
The positive relationship between (Y + Zr) values, measured three months after hysteroscopic metroplasty, and Z (**A**) and (Y + Z) (**B**) values measured before surgery.

At the three months follow-up, Zr exceeded 50% of Y in forty-three patients (72%) who underwent a second step of metroplasty in the early follicular phase of the following menstrual cycle, because Y + Zr was greater than 1 cm, allowing the possibility of a further correction without substantially reducing the thickness of the fundal myometrial wall. Conversely, an optimal removal of the intrauterine septum was confirmed in seventeen patients (28%). As shown in Figure 5, at the three months ultrasound follow-up, the absolute change in (Y + Zr) was positively correlated with the Z and (Y + Z) values before surgery.

The ultrasonographic evaluation of the uterine fundus before surgery showed a concave morphology in thirteen cases (21.6%), convex in thirteen (21.6%) and flat in thirty-four patients (56.6%). The uterine fundal morphology appeared modified in thirty-five cases (58%) after metroplasty. Instead, twenty-five patients (42%) had the same shape of the uterine fundus (either convex or flat) both in the pre-surgical evaluation and after three months (Table 2).

The test for marginal homogeneity indicated a significantly different distribution of the uterine fundus shape three months after surgery (test statistic =32.54, *p* < 0.001).

**Figure 6:**
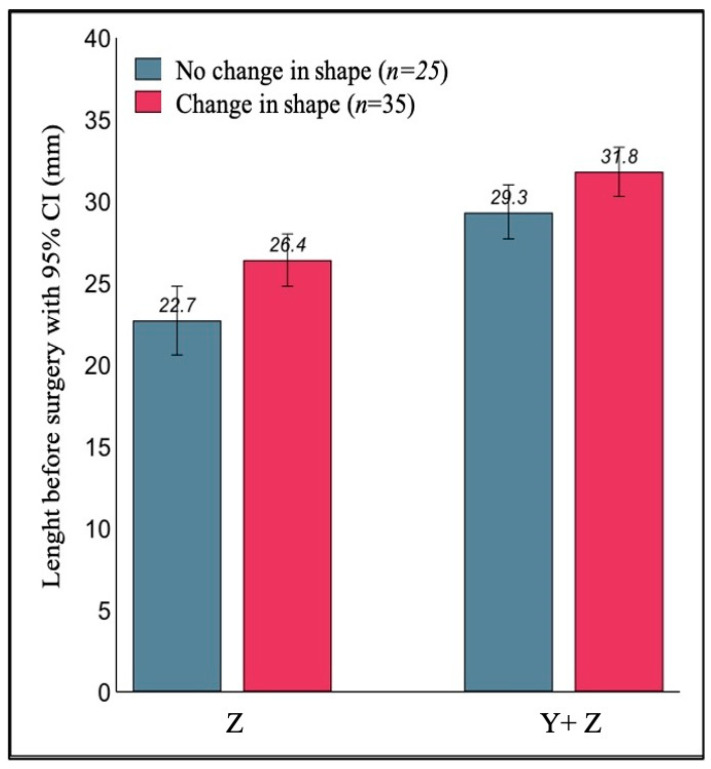
Mean Z and (Y + Z) values measured before surgery and their relationship with the change of the fundal uterine shape at three months after surgery.

As shown in Figure 6, the thirty-five women who showed a change in the shape of the uterine fundus had a significantly longer septum before surgery, in fact compared to the twenty-five women with no change in shape, Z and (Y+Z) were on average 3.7 mm longer (95% CI: 1.1–6.2; *p* = 0.005), and 2.5 mm longer (95% CI: 0.2–4.7; *p*= 0.031), respectively.

Moreover we also analyzed, separately, the results of the subsample of patients scheduled for the second step of surgery (*n* = 43). Fifteen patients (35%) had the same shape of the uterine fundus at both ultrasound evaluations (convex in six cases and flat in nine cases) (Table 3).

**Figure 7:**
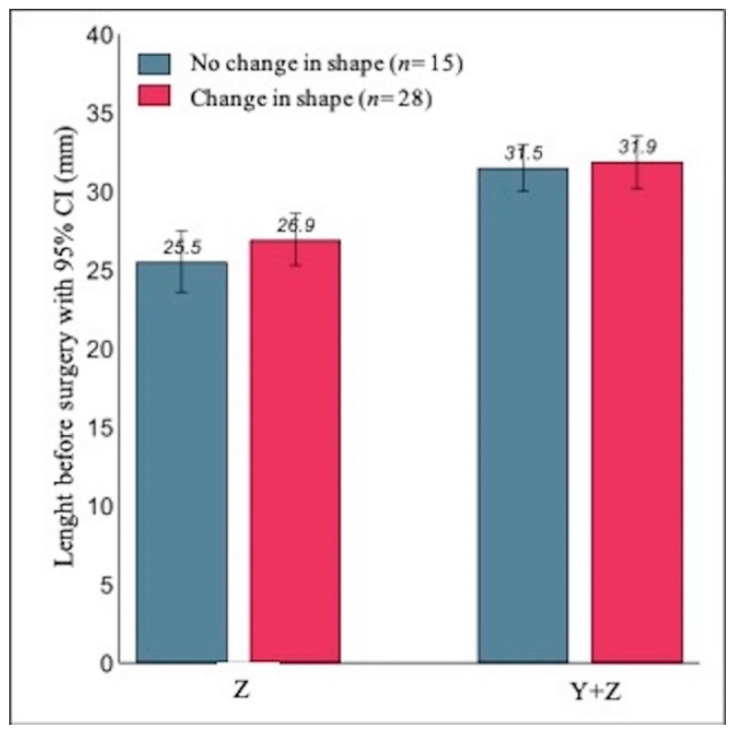
Mean Z and (Y + Z) values measured before surgery and their relationship with the change of the fundal uterine shape at three months after surgery in patients scheduled for second surgery (*n* = 43).

In addition, in this subsample, the test for marginal homogeneity indicated a significantly different distribution of the shape of the uterine fundus at the three months ultrasound follow-up (test statistic = 26.51, *p*-value < 0.001). As shown in Figure 7, the mean length of the septum before surgery in the 28 patients who exhibited a change in the shape of the fundus was not significantly different from the mean length in the 15 patients with no change in shape. More specifically, Y was on average 1.4 mm longer (95% CI: −1.2 to 4.0; *p*-value = 0.278), and (Y + Z) was on average 0.4 mm longer (95% CI: −2.0 to 2.8; *p*-value = 0.748).

## 4. Discussion

The septate uterus is the most common congenital uterine malformation and the hysteroscopic metroplasty is considered the treatment of choice for women with desire for pregnancy [28,29,30]. Nevertheless, data on improvement of the reproductive outcomes after hysteroscopic metroplasty are contradictory and limited.

In our study, a systematic ultrasonographic evaluation prior to and after metroplasty, performed with the Di Spiezio technique, showed a significative remodeling of the inner and outer profiles of the uterus at three months after surgery. This led to a diagnosis of a residual septum in 72% of the patients, for which a second step metroplasty was necessary. Moreover, we recorded a significative change in the external shape of the fundus in 35% of the patients.

Our results confirm that Di Spiezio’s metroplasty technique allows for an initial complete removal of the uterine septum, as proved by early post-operative ultrasound. However, at the three-months follow up ultrasound there was a thickening of the fundal myometrial wall due to an increase in Zr and Y values. This resulted in an indication for a second-step metroplasty for 72% of the patients; otherwise, the patients were not treated adequately. We believed that the delayed increase in Zr and Y values may be due to the dynamic capabilities of the fundal uterine wall determined by its myometrial component. This has led to changes in both the external and internal profiles over time and not as a direct and immediate consequence of the hysteroscopic procedure.

According to Rikken et al. [2], the histological composition of the septum does not differ to that of the normal uterine wall, made of myometrium and endometrium. So, we believe that the dynamic changes following metroplasty could be related to uterine contractions triggered by the section of muscle fiber cells in the septum itself. Furthermore, our outcomes showed that major uterine remodeling has occurred in cases with longer uterine septa.

The prospective design, the homogeneity of the population studied, the use of a standardized metroplasty technique and a systematic ultrasound examination according to the CONUTA criteria, represent the strengths of our study. We used the 3D-TVS to evaluate not only the morphology of the uterine cavity, but also the external profile of the uterine fundus, before and after the hysteroscopic metroplasty, and recorded all of the changes that occurred in the three months of follow-up after surgery.

The limitations of our study are the small number of patients included, not having performed a control ultrasound after the second step of metroplasty, proposing more surgical procedures to infertile patients despite the dubious utility in improving fertility and the lack of data on reproductive outcomes. For these reasons, we are continuing to enroll patients, collect and analyze data that will be the subject of a subsequent study.

This work reinforces the idea of uterine remodeling after hysteroscopic metroplasty [25,26], due to the intrinsic histological characteristics of the septum, and the ability of the myometrium to modify the shape and thickness of the uterine walls not only in physiological conditions such as pregnancy, but also iatrogenic, such as surgery.

Based on our outcomes, it seems appropriate to offer a follow-up ultrasound to all patients undergoing hysteroscopic metroplasty at least three months later, even if this was performed by an expert surgeon and using a standardized technique. The ultrasound examination will be decisive in identifying patients deserving of a second step metroplasty to restore a regular morphology of the uterine.

We believe that our findings could be considered a novelty and, therefore, recommend prospective controlled studies with larger cohorts of patients and longer follow-ups to confirm our data, to observe possible uterine remodeling after second step metroplasty and to evaluate reproductive outcomes.

## Figures and Tables

**Table 1:** Characteristics of women included in the study.

Characteristics	Values
Age (years ± SD)	30.7 ± 4.18
Indications for surgery, *n* (%)	
Primary infertility	47 (78.3)
Spontaneous abortions	13 (21.6)
Type of uterine septum	
Partial septum	29 (48.3)
Complete septum	31 (51.6)
Length of the septum, Z (mm ± SD)	24.9 ± 5.13
Thickness of uterine myometrial fundus, X (mm ± SD)	5.9 ± 1.94

**Table 2:** Shape of the uterine fundus before surgery and at three-month evaluation after surgery.

Uterine Fundus Before Surgery *n* (%)	Uterine Fundus at Three Months Follow-Up			All
	Concave	Convex	Flat	
Concave	0 (0%)	4 (7%)	9 (15%)	13 (22%)
Convex	0 (0%)	13 (22%)	0 (0%)	13 (22%)
Flat	0 (0%)	22 (36%)	12 (20%)	34 (56%)
All	0 (0%)	39 (65%)	21 (35%)	60 (100%)

**Table 3:** Shape of the uterine fundus before surgery and at three-months evaluation after surgery in patients scheduled for a second-step surgery (*n*= 43).

Uterine Fundus before Surgery *n* (%)	Uterine Fundus at 3-Month Follow-Up			All
	Concave	Convex	Flat	
Concave	0 (0%)	2 (5%)	9 (21%)	11 (26%)
Convex	0 (0%)	6 (14%)	0 (0%)	6 (14%)
Flat	0 (0%)	17 (40%)	9 (21%)	26 (60%)
All	0 (0%)	25 (58%)	18 (42%)	43 (100%)

## Data Availability

The data presented in this study are available on request from the corresponding author. The data are not publicly available due to the complexity of method of data storage.
